# A High-Resolution Open Source Platform for Building Envelope Thermal Performance Assessment Using a Wireless Sensor Network

**DOI:** 10.3390/s20061755

**Published:** 2020-03-21

**Authors:** Romwald Lihakanga, Yuan Ding, Gabriela M. Medero, Samuel Chapman, George Goussetis

**Affiliations:** 1 Institute for Infrastructure and Environment, Heriot Watt University, Edinburgh EH14 4AS, UK; 2 Institute of Sensors, Signals and Systems, Heriot Watt University, Edinburgh EH14 4AS, UK

**Keywords:** heat flow, open platform, thermal performance, wireless sensor network

## Abstract

This paper presents an in-situ wireless sensor network (WSN) for building envelope thermal transmission analysis. The WSN is able to track heat flows in various weather conditions in real-time. The developed system focuses on long-term in-situ building material variation analysis, which cannot be readily achieved using current approaches, especially when the number of measurement hotspots is large. This paper describes the implementation of the proposed system using the heat flow method enabled through an adaptable and low-cost wireless network, validated via a laboratory experiment.

## 1. Introduction

The thermal performance of building materials is an important factor that greatly affects a building’s energy performance and efficiency, i.e., it is used for the energy auditing of new and existing buildings [1,2,3]. It describes how building materials respond to the ambient environment in the daily or seasonal cycle. It directly relates to the thermal comfort of the building occupants, as well as the energy consumed for heating or cooling [1,4,5,6,7].

The thermal performance of building materials can be characterized using thermal transmittance (U-value), thermal resistance, thermal conductivity, and diffusivity [8]. Measurements can be done in a laboratory (ex-situ) or on-site (in-situ). The hot box method [9] is commonly used in a lab environment, while the heat flow method has been recommended in the BS ISO 9869 [10] for in-situ measurements. Other in-situ approaches, such as infrared (IR) thermography [11] and calculation methods [12], have also been developed to evaluate thermal transmittance and thermal resistance. To present a technological background, some widely used methods for the thermal performance evaluation of in-situ measurement (the focus of this paper) are summarized below, together with their advantages and disadvantages.

The heat flow method, described in BS ISO 9869 [10], has been widely employed to measure the thermal transmission properties of building structures that consist of homogeneous layers perpendicular to the heat flow. The existing measurements, as per BS-9869-1, use offline and hardwired data acquisition equipment. One unit is located indoors to measure the air temperature, surface temperature, and heat flux through the material, while the other unit is located outdoors to measure the air and surface temperature. Each unit measures and stores data separately, before being transferred centrally for further analysis. In BS 9869-1, this is the only recommended electrical method for the in-situ measurement of thermal performance parameters. The properties that can be measured include:

- Thermal resistance and conductivity from the surface to surface of both sides of the material.
- Thermal transmittance from the environment to the environment of both sides of the material.

For this method to give acceptable thermal transmission estimations, the following factors (or assumptions) have to be carefully examined [10]:

- The thermal properties of the materials being tested and the heat transfer coefficient need to be constant over the entire measurement region.
- The heat stored in the materials is negligible compared to the amount of heat that moves through the materials.
- The temperature difference between indoors and outdoors is at least 10 ℃.
- The wind speed is less than 3 m/s.
- The heat flow sensor is shielded against direct exposure to solar radiation and moisture.

Even if the above requirements have been met, to date, the current measurement systems exploiting this method still have the following limitations [13,14]:

- The measurements are non-continuous and non-real-time since the collected data have to be processed offline. Thus, any inconsistency in measurements cannot be observed and efficiently corrected during the measurement.
- The equipment is bulky, hardwired, and consumes a significant amount of power.

The calculation method stipulated in ISO 6496 [12] exploits an electrical analogy of the building materials when they are assumed to be in steady-state conditions. For given material properties, e.g., thickness and thermal conductivity, thermal transmittance can be approximately derived. However, these estimations are not accurate because of the high uncertainty of material properties, and the uncontrolled inconsistence between lab and on-site environments [12].

Infrared (IR) thermography is one of the most popular non-destructive testing technologies for building material analysis. It measures temperature via thermal radiation and produces images that represent the detected infrared energy of the target object. The temperature distribution on the object is illustrated with different colors, which can then be analyzed to extract material information and abnormalities. The accuracy of IR measurements highly depends on the measurement distance from the target objects, wind speed, atmospheric particles, and ambient temperature [15,16].

Despite its tolerable accuracy, the IR method suffers from the following disadvantages:

- Image interpretation is usually challenging.
- Proper environmental conditions have to be met to ensure that external factors, such as wind, solar radiation, and moisture, do not interfere with the measurements.
- It cannot be easily integrated within other platforms.

A three-temperature measurement methodology was developed and used in [13,14]. In this approach, three temperatures, indoor surface temperature, indoor air temperature, and outdoor air temperature, are measured and used to evaluate material thermal performance. However, these analyses are limited only on thermal transmittance measurements for energy efficiency evaluation purposes. It has not been demonstrated how the method can be used to measure thermal conductivity and thermal resistance. Though this method has shown some promising results, it is still at the research-level and cannot be used for reference measurements. More studies on the effects of material thermal mass and the homogeneity of measurements are needed before this method can be adopted.

The motivation behind this paper is to address the challenges facing the existing building thermal performance measurements in BS 9869-1. In this work, the BS 9869-1 method was expanded using custom-designed high-resolution sensor nodes that are able to wirelessly sense, process, and send thermal performance parameters in a real-time fashion. This measurement platform enjoys a high resolution and a low-cost. The dataset obtained by the proposed system is then used to analyze how building materials’ thermal performance varies when interacting with environmental thermal variations in daily or seasonal cycles. The designed platform adopts the heat flow method. The heat flux is measured together with the surface and air temperature on both sides of the structure being tested. The system allows multiple measurements to be taken wirelessly, and this open source platform can be readily expanded and adjusted as per the user’s requirements. After being installed and configured, it operates without the need for human intervention. The system also incorporates a database to store the measured results and graphical interface for visualization and data analysis.

## 2. Thermal Performance Measurement

**Figure 1:**
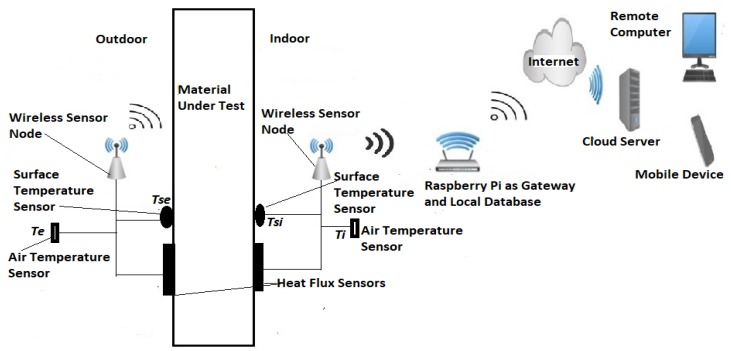
Proposed measurement system architecture.

The heat flow method was customized (see the proposed system architecture in Figure 1) to meet the requirements stated in the British Standard [10] with reduced power consumption. The following parameters were measured by the selected sensor nodes and sent wirelessly to the gateway for processing through Xbee communication modules.

- Ambient air temperature on both sides of the walls, noted as *T_e_* and *T_i_* with units of K (Kelvin)
- The surface temperature on both sides of the walls, noted as *T_se_* and *T_si_* with units of K (Kelvin)
- Heat flux through the walls (envelope), whose density is noted as *Φ* with units of W/m^2^.

Direct computation (average method) is employed in the analysis of estimating thermal transmittance (*U-Value* in (1) with units of W/(m^2^·K)), thermal resistance (*R-Value* in (2) with units of m^2^·K/W), and thermal conductivity (*Λ-Value* in (3) with units of W/(m^2^·K)), where index *j* refers to the *j^th^* measurement spots out of *J*. This method assumes a steady-state heat flow and safely neglects the thermal mass of the material [10]. The factors and setup in the conventional method [10] have been taken into account for Equations (1)–(3) to give close approximate values:

(1) $$U-Value=\frac{\sum_{j=1}^{J}\Phi_{j}}{\sum_{j=1}^{J}\left(T_{ij}-T_{ej}\right)}$$

(2) $$R-Value=\frac{\sum_{j=1}^{J}\left(T_{sij}-T_{sej}\right)}{\sum_{j=1}^{J}\Phi_{j}}$$

(3) $$\Lambda -Value=\frac{\sum_{j=1}^{J}\Phi_{j}}{\sum_{j=1}^{J}\left(T_{sij}-T_{sej}\right)}.$$

## 3. System Description

### 3.1. Sensor Nodes

**Figure 2:**
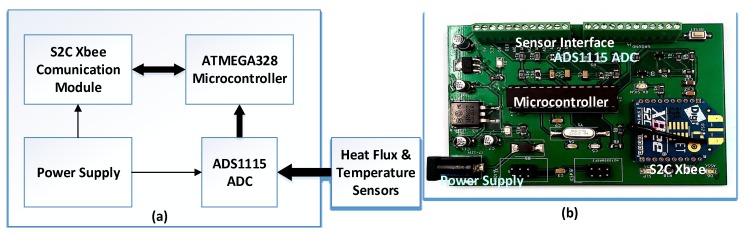
Sensor node, (**a**) block diagram, and (**b**) prototype.

Each sensor node in Figure 2 is a bespoke design for a low-power purpose, consisting of a power supply unit, a high-resolution ADS1115 16-bit ADC [17], an Atmeg328P-PU microcontroller [18], and an S2C Xbee wireless communication module based on the IEEE 802.15.4/ZigBee Standard [19]. The Xbee module was selected because of its small form factor, low power, and low cost. The ATmega328P-PU was used for its easy interface with the Xbee module and its simple programming in C using the Arduino open-source software. The ADS1115 ADC was added to provide enough resolution for the heat flux sensor output, which has a voltage swing less than 5 µV. ADS1115 can be configured as four single-ended input channels or two differential-input channels. As ADS1115 uses the I2C bus, it allows easy expansion of the system if more high-resolution channels are needed. These nodes can be powered by a button battery. The power consumption of the nodes can be further minimized by sending sensing data in a duty cycle mode, allowing the communication modules to enter deep sleep mode for most of the time.

Sensor nodes read the data from the connected sensors, converting them into the required format before sending the data wirelessly through the communication module. The described sensor node design allows the following to be achieved:

- More sensors can be added into the sensor nodes without additional hardware in the gateway node. Only minor software modifications are required.
- More sensor nodes using the same communication protocols can be deployed.
- The setup and calibration for all nodes are performed and stored locally.

### 3.2. Gateway/Coordinator Node

**Figure 3:**
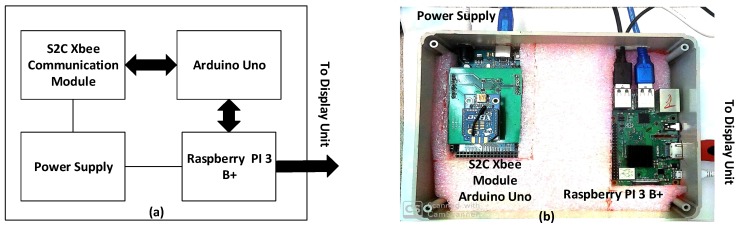
Gateway node, (**a**) block diagram, and (**b**) prototype.

A gateway or coordinator is employed in the platform to wirelessly receive sensor data from different sensor nodes located indoors and outdoors of the building and to pre-process, store, and relay the data. The gateway setup in Figure 3 consists of an S2C Xbee module [19] connected to the Arduino board for relaying and pre-processing the data before being sent to a Raspberry Pi module 3B+ [20]. A database and a configured webserver are also created in the gateway to permit the data to be accessed locally and remotely. For administration of MYSQL over the Web, phpMyAdmin has been used. This is a free software package that is used to manage databases, tables, columns, and user permissions. It allows data to be exported in various formats and to create graphics of the database in numerous formats [21].

## 4. Experiment Setup and Results

**Figure 4:**
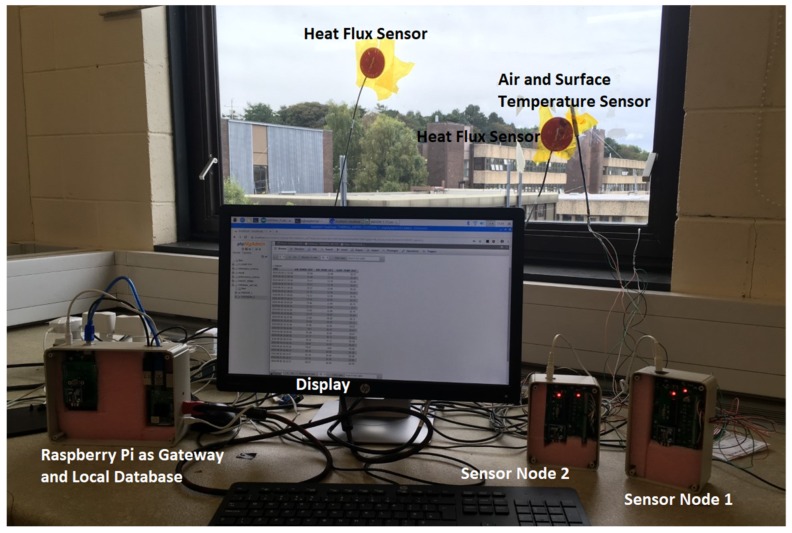
Measurement setup of the proposed platform.

To validate the designed system, sensors were installed to measure the thermal transmission value *U* on one of the windows in the lab, as shown in Figure 4. The measurements of heat flux and indoor and outdoor temperatures (surface and ambient) were collected continuously for seven days. The sensor HFP01-05 from Hukseflux with a sensitivity of 62.2 × 10^-6^ V/(W/m^2^) (recommended in the BS Standard [10]) was chosen for heat flow measurement and was initially calibrated by the manufacturer. For surface temperature measurements, the sensors used included an NTC thermistor model B57020M2 from EPCOS that is highly resistant to water and moisture. HTM2500LF [22] was used to measure the air temperature on both sides of the structure being tested. This sensor is stable, highly sensitive, and unaffected by water immersion. Before the measurement campaign, the heat flux sensors were tested/validated in the lab using a high precision millivoltmeter, and the temperature sensors were calibrated in a controlled lab environment using a Kambic climatic chamber.

To equip the wireless capability of the developed system, two wireless sensor nodes were used in the experiment. One wireless node was placed outside of the window to measure the outdoor air temperature and the outdoor glass surface temperature. The other node was placed indoors to measure the heat flow through the structure, as well as the air temperature and surface temperature of the structure being tested. Because of the size of the network, the star network topology was selected and used for communication between the sensor nodes and the gateway.

Other reasons for selecting the star network at this stage of the design include the following:

- When one node fails, it will not affect other parts of the network.
- It facilitates adding or removing nodes in the network.
- Easy monitoring of the network since all nodes are managed by the gateway node.

Despite the above benefits, the star network has the following drawbacks:

- More channels are required because each node is connected directly to the gateway.
- Since the entire network depends on the gateway, if the gateway fails, the entire network goes down.
- The capacity of the gateway determines the performance and number of nodes that can be accommodated by the network.

The data were collected every five minutes and then sent to the gateway node for pre-processing before being pushed into an SQL server. For the measurement results to be valid, the conditions/requirements stipulated in the BS 9869-1 [10] guidance were established during the setup of the experiment and tracked for the entire duration of the measurement.

**Figure 5:**
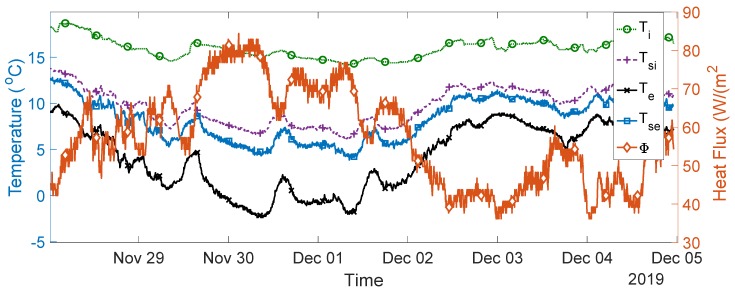
Overall thermal transmission parameter measurement data for 7 days.

**Figure 6:**
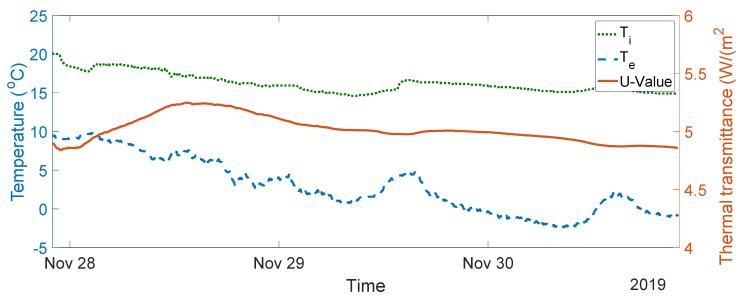
Thermal transmittance measurement data from Day 1 to Day 3. (W/(m^2^K)).

**Figure 7:**
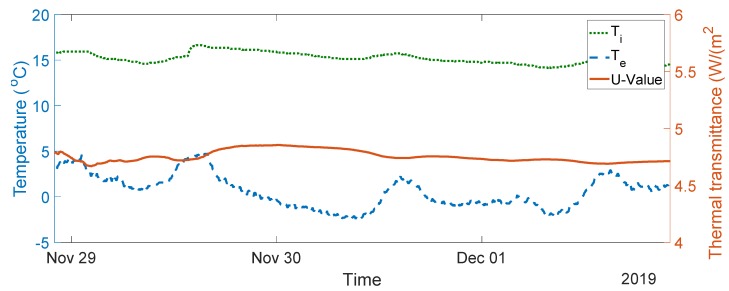
Thermal transmittance measurement data between Day 2 and Day 4.

**Figure 8:**
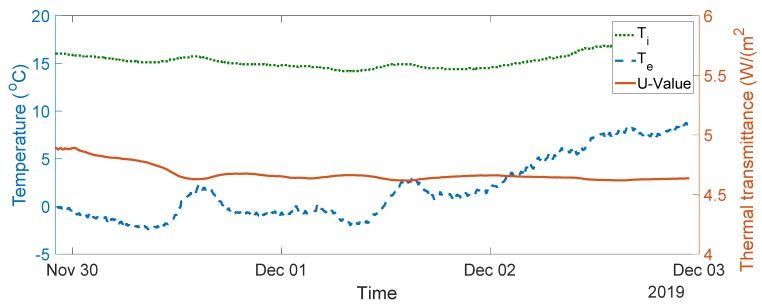
Thermal transmittance measurement data between Day 3 and Day 5.

**Figure 9:**
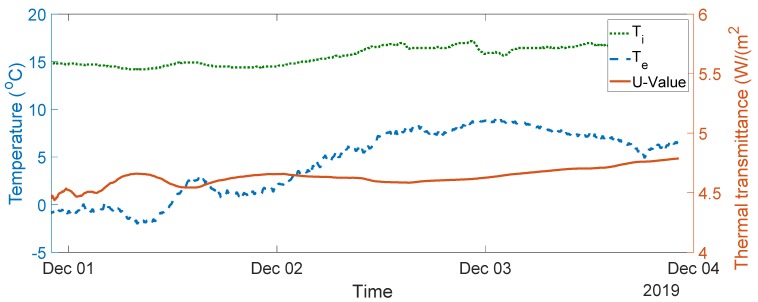
Thermal transmittance measurement data between Day 4 and Day 6.

**Figure 10:**
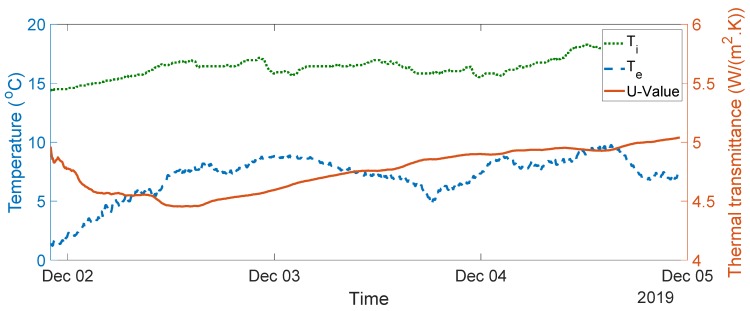
Thermal transmittance (U-value) measurement data between Day 5 and Day 7.

The seven-day measurement data are plotted in Figure 5. The average indoor–outdoor air temperature difference over the entire duration of the measurement campaign was slightly below 10 °C, i.e., 9.64 °C. For the data used in the final analysis, the average indoor–outdoor air temperature difference was greater than 10 °C, the wind speed measured was less than 3 m/s, and there was no direct solar radiation on the sensors (as the window faces north). To validate the designed system, thermal transmittance, thermal resistance, and thermal conductivity were calculated through (1) to (3) over three consecutive days, as advised in the British Standard [10]. The measurements and range of the typical U-values are shown in Figure 6, Figure 7, Figure 8, Figure 9 and Figure 10 and summarized in Table 1. The thermal performance value of the window glass being tested was taken from [23,24].

**Figure 11:**
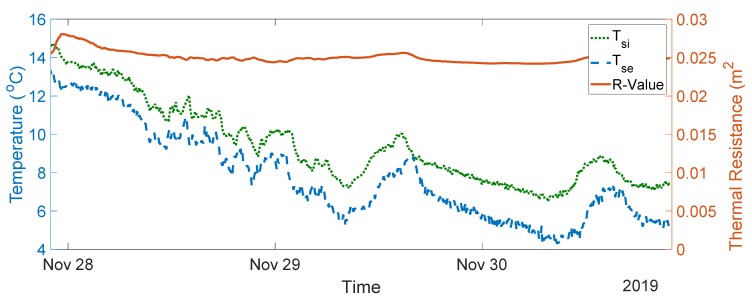
Thermal resistance measurement data from Day 1 to Day 3.

**Figure 12:**
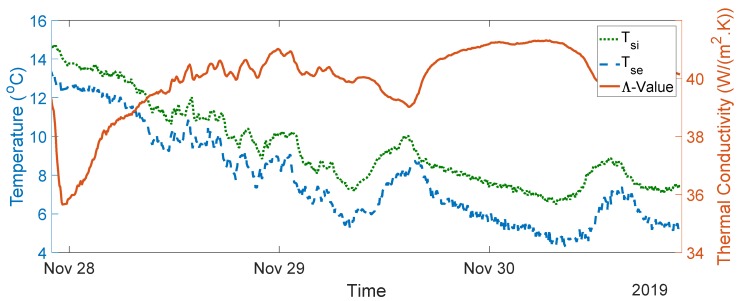
Thermal conductivity measurement data from Day 1 to Day 3.

Based on the seven-day measurements summarized in Table 1, Day 1 to Day 3 and Day 5 to Day 7 were relatively steady. Using the steady-state values from the measurements of Day 1 to Day 3, the average values of thermal resistance and thermal conductance were also evaluated. The average thermal resistance value was 0.025 W/(m^2^K), as shown in Figure 11**,** while the average value of thermal conductivity was 40 W/(m^2^K), as shown in Figure 12.

The single glazed window used in the experiment was estimated to have a typical U-value ranging between 4.8 and 5.8 W/m^2^K and a thermal resistance value of 0.025 m^2^·K/W [23,24]. In the case of the real-time measurements obtained, the thermal transmittance values were between 4.55 and 5.11 W/m^2^K, and the average thermal transmittance value for the measurement was 4.82 W/m^2^K. The measured value of thermal resistance was between 0.02 and 0.03 m^2^·K/W, with an average value of 0.025 m^2^·K/W. The values obtained are well within the range of the typical values provided.

## 5. Discussion

It is important to measure and analyze thermal performance to understand the energy performance of building materials and how the materials interact with building occupants and the environment, as well as to better predict the energy efficiency of buildings. The conventional heat flow method has been recommended by the British Standards for the in-situ measurement and analysis of the thermal transmission parameters of the building materials and structures. However, to date, the use of this system is limited and impractical when the number of measurement hotspots is large and located at different locations. The designed and implemented platform in this paper addresses the limitations of the conventional heat flow meter method by designing a system with the following features:

- The system is able to collect and process data in real-time.
- The system can be customized to take any number of measurements with any time interval, as per the requirements of the user or the specifications of the measurement.
- Using the same gateway (the coordinator node), different measurements within the distance of 100 m indoors or 1.2 km outdoors can be accommodated.
- These measurements can be accessed remotely in any place through the Internet, and the gateway can also be reconfigured remotely when needed.
- A connection to the Internet can be easily initiated through Wi-Fi or a cable connection.
- The developed system is accurate, cheap, and small in size.

## 6. Conclusions

Demands for the in-situ thermal performance measurement and an analysis of building materials and structures are increasing dramatically, in the search for tools, equipment, and methods that are flexible, low cost, and accurate. While a heat flow meter has been recommended as standard equipment for in-situ measurements of thermal transmission parameters, it is expensive, non-continuous, and impractical when a large number of measurement hotspots are involved.

This paper has shown how the limitations and data lacunae of conventional heat flow meters can be eliminated by designing a high-resolution, open-source platform for building envelope thermal performance measurement and analysis. The designed platform uses the same principle as the heat flow meter but with increased flexibility and enhanced performance. The system is an open platform with a high resolution and low-cost. In addition, it is compact, easy to customize, and easy to deploy and maintain. Another important feature of the system is that the gateway, comprising an embedded system (i.e., a Raspberry Pi), houses a database server and webserver, endowing the system with the capability to store, process, and allow remote access to the information. The system can be extended from the star network to the mesh network when more sensing nodes are added in different rooms or buildings. Depending on the requirements of the measurements, the power consumption per node can be further reduced by adding a high side loading switching and operating microcontroller in duty cycle mode. Without jeopardizing the quality of BS 9869-1 measurements, any other energy efficient–saving approach can be applied.

## Figures and Tables

**Table 1:** Summary of the thermal transmittance measurements.

Measurement Period (Day)	Typical U-Value (W/m^2^K) [22,23]	U-Value Measurement (W/m^2^K)	Average U-Value Measurement (W/m^2^K)
1–3	4.8–5.8	4.56–4.86, see Figure 6	4.82
2–4		4.55–4.78, see Figure 7	4.75
3–5		4.55–4.65, see Figure 8	4.65
4–6		4.64–4.79, see Figure 9	4.75
5–7		4.55–5.11, see Figure 10	4.85
